# Huaier Attenuates Renal Injury in Type 2 Diabetic Nephropathy by Ameliorating Mitochondrial Dysfunction and Inhibiting Tubular Epithelial Cell Apoptosis

**DOI:** 10.1111/1753-0407.70290

**Published:** 2026-10-29

**Authors:** Yucan Guan, Xiaoying Bai, Xianping Wei, Tao Ding, Ping Luo, Ping Nie

**Affiliations:** ^1^ Department of Nephrology The Second Hospital of Jilin University Changchun Jilin Province China; ^2^ Clinical Research Department The Second Hospital of Jilin University Changchun Jilin Province China; ^3^ Center for Pharmacodynamics and Toxicology Evaluation Jilin Academy of Chinese Medicine Sciences Changchun Jilin Province China

**Keywords:** apoptosis, diabetic nephropathy, Huaier, renal protection, Wnt5a/β‐catenin pathway

## Abstract

**Background:**

Diabetic nephropathy (DN) is a major microvascular complication of Type 2 diabetes, with renal tubular epithelial cells (RTECs) apoptosis playing a pivotal role in its progression. The aqueous extract of *Trametes robiniophila* Murr. (Huaier) has shown renoprotective effects, but its specific molecular mechanisms in DN remain to be fully elucidated.

**Objective:**

This study aimed to investigate the therapeutic effects of Huaier aqueous extract on DN and verify whether its anti‐apoptotic mechanism is mediated through the Wnt/β‐catenin pathway using the pathway activator SKL2001.

**Methods:**

In vivo, KKAy Type 2 diabetic mice were treated with Huaier (5 g/kg/day) for 12 weeks to evaluate renal function and histopathology. In vitro, HK‐2 cells were treated with high glucose and palmitate (HG + PA) to induce injury. The Wnt/β‐catenin pathway activator SKL2001 was employed to determine if activating this pathway could abolish the protective effects of Huaier.

**Results:**

Bioinformatics analysis indicates that the regulation of apoptosis is the primary mechanism by which Huaier improves DN. In vivo, Huaier reduced UACR and improved renal histopathology in KKAy mice. Consistently, TUNEL staining and molecular analysis confirmed that Huaier significantly suppressed RTECs apoptosis within the renal tissues. In vitro experiments demonstrated that Huaier significantly inhibited HG + PA‐induced oxidative stress, mitochondrial dysfunction, and RTECs apoptosis. Notably, the administration of SKL2001 effectively reversed these protective effects, leading to increased β‐catenin expression and elevated apoptosis levels despite Huaier treatment.

**Conclusion:**

Huaier aqueous extract mitigates RTECs apoptosis and renal injury in Type 2 diabetic nephropathy by suppressing the Wnt5a/β‐catenin signaling pathway.

## Highlights

- Huaier aqueous extract ameliorates renal dysfunction and histopathological injury in Type 2 diabetic nephropathy.
- Huaier ameliorates mitochondrial dysfunction, oxidative stress, and apoptosis in HK‐2 cells.
- The renoprotective effects of Huaier are mediated by inhibition of aberrant Wnt5a/β‐catenin signaling.

## Graphical abstract

Huaier aqueous extract significantly attenuates renal injury and tubular epithelial cell apoptosis in Type 2 diabetic nephropathy. This protective mechanism is driven by the amelioration of mitochondrial dysfunction and the targeted suppression of the aberrant Wnt5a/β‐catenin signaling pathway.

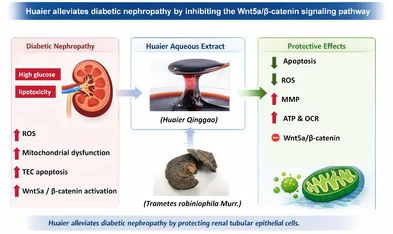

**Abbreviations**
ACEIs: angiotensin‐converting enzyme inhibitors
ARBs: angiotensin II receptor blockers
BP: biological processes
CC: cellular components
DL: drug‐likeness ≥ 0.1
DN: diabetic nephropathy
ESRD: end‐stage renal disease
GBM: glomerular basement membrane
GO: gene ontology
HMGB1: high‐mobility group box 1
HQH: Huaiqihuang
IgN: IgA nephropathy
KEGG: Kyoto encyclopedia of genes and genomes
MF: molecular functions
MMP: mitochondrial membrane potential
OB: oral bioavailability
PNS: primary nephrotic syndrome
ROS: reactive oxygen species
Scr: serum creatinine
TCM: traditional Chinese medicine
TEM: transmission electron microscopy
UACR: urinary albumin‐to‐creatinine ratio

## 1 Introduction

Diabetic nephropathy (DN) constitutes a predominant microvascular complication of diabetes mellitus. DN severely compromises patient quality of life and significantly escalates the risk of end‐stage renal disease (ESRD) and cardiovascular sequelae [1, 2]. Studies have reported that the pathogenesis of DN is closely associated with multiple factors, including hyperglycemia, oxidative stress, inflammatory responses, and apoptosis [3, 4, 5]. Apoptosis constitutes a fundamental mode of programmed cell death indispensable for developmental processes and the maintenance of physiological homeostasis [6]. However, in the pathological context of DN, research focus has increasingly expanded from glomerular injury to the critical role of renal tubular epithelial cells (RTECs). Due to their high metabolic activity and oxygen demand, proximal RTECs are particularly vulnerable to the oxidative stress and inflammatory milieu induced by hyperglycemia [7, 8]. Consequently, aberrant RTECs apoptosis drives tubulointerstitial fibrosis, accelerating the progression to ESRD. Therefore, targeting RTECs apoptosis represents a vital therapeutic strategy.

Recently, Traditional Chinese Medicine (TCM) has exhibited distinct therapeutic advantages in the clinical management of DN. Characterized by their multicomponent and multitarget properties, TCM formulations exhibit significant potential in intervening in RTECs apoptosis by regulating mitochondrial function, inhibiting endoplasmic reticulum stress, and blocking death receptor pathways [7, 9, 10]. Among the promising candidates, *Trametes robiniophila* Murr., a medicinal fungus traditionally recorded in the Tang Materia Medica (Tang Ben Cao) [11], has garnered attention for its diverse biological activities. *Trametes robiniophila* Murr., predominantly colonizing the trunks of Sophora japonica [12], is extensively utilized in clinical practice for its anti‐tumor and immunomodulatory properties; notably, emerging evidence has indicated its potential renoprotective effects. The aqueous extract of *Trametes robiniophila* Murr. (Huaier) is enriched with diverse bioactive constituents—notably polysaccharides and glycoproteins—which confer robust anti‐inflammatory, antioxidant, and renoprotective properties [13, 14]. Modern preparations derived from this fungus have been integrated into clinical practice. Huaier granule, a preparation made from the aqueous extract of *Trametes robiniophila* Murr., is approved for marketing by the National Medical Products Administration in China. Huaier granule is widely used as an adjuvant therapy for various malignant tumors, such as hepatocellular carcinoma, bladder cancer, and stomach cancer, and it has both tumor‐suppressing and immune‐enhancing properties. Together with other formulations such as Huaier ointment, these preparations are broadly applied in the treatment of both tumors and kidney diseases. Huaiqihuang (HQH), a compound formulation with Huaier as its principal component, has exhibited robust therapeutic efficacy in the management of diverse renal pathologies, including IgA nephropathy, primary nephrotic syndrome, and chemotherapy‐induced nephrotoxicity [13, 15]. Both in vivo and clinical studies have demonstrated a favorable safety profile of Huaier [16]. However, the application of Huaier in the context of DN remains largely unexplored.

The present study systematically investigated the pharmacological effects and underlying mechanisms of Huaier in DN through in vitro and in vivo experiments, providing a theoretical basis for its potential clinical application.

## 2 Materials and Methods

### 2.1 Preparation of Huaier Aqueous Extract

Huaier aqueous extract (Huaier Qinggao, hereinafter referred to as Huaier) used in this study is a commercially available standardized preparation obtained from Qidong Gaitianli Pharmaceutical Co. Ltd. (Jiangsu, China). The aqueous extract is produced through a patented and standardized manufacturing process based on hot‐water extraction of Huaier fungal material followed by concentration and purification. For experimental use, the Huaier extract was dissolved in culture medium, sterilized by filtration through a 0.22 μm membrane, and prepared as a stock solution. The stock solution was stored at 4°C and diluted to the required concentrations immediately before use. The Huaier aqueous extract (Huaier Qinggao) was characterized through molecular weight distribution analysis using HPLC‐GPC‐ELSD and chromatographic fingerprinting (Supplementary Figure S1 and Supplementary Table S1), which confirmed that the extract is predominantly enriched in macromolecular polysaccharides and proteoglycans.

### 2.2 Cell Culture and Treatment

Human renal tubular epithelial cells (HK‐2 cells), a kind gift from the Laboratory of Nephrology, University of Louisville, were cultured in DMEM/F12 (HyClone, Cytiva, China) supplemented with 8% fetal bovine serum and 1% penicillin–streptomycin under standard conditions (37°C, 5% CO_2_). To simulate DN, cells were exposed to 30 mM glucose (24 h) followed by 300 μM palmitate (6 h) [17]. Treatment groups were exposed to graded concentrations of Huaier for 24 h. The concentration of SKL2001 (MCE, HY‐101085), a β‐catenin agonist, was 20 μM.

#### 2.2.1 Cell Viability Assay

To evaluate cytotoxicity, HK‐2 cells were cultured in 96‐well plates (8 × 10^3^ cells/well) and incubated with graded concentrations of Huaier for durations of 24 or 48 h. Following the intervention period, cell viability was assessed by adding 10 μL of CCK‐8 reagent (Absin, China) to each well for a 1–2 h reaction period. The resulting metabolic activity was quantified by measuring optical density (OD) at 450 nm.

#### 2.2.2 Intracellular ROS Detection

Intracellular ROS levels were detected using a 10 μM DCFH‐DA probe (Beyotime, China). Following a 20‐min dark incubation at 37°C, fluorescence signals (Ex/Em: 488/525 nm) were captured and analyzed via ImageJ.

#### 2.2.3 Mitochondrial Membrane Potential Assay

Mitochondrial membrane potential (MMP) was assessed via JC‐1 staining (Solarbio, China). Following a 20‐min dark incubation at 37°C with the JC‐1 working solution, cells were washed and imaged via fluorescence microscopy to monitor red/green fluorescence shifts.

#### 2.2.4 Apoptosis Assay

Apoptosis was qualitatively assessed via fluorescence microscopy using an Annexin V‐FITC/PI kit (Yeasen, China). Posttreatment, samples were stained with 5 μL of Annexin V‐FITC and 10 μL of PI (15 min, dark, room temperature) to visualize early and late apoptotic stages. Quantitatively, apoptosis rates were determined using the eBioscience Annexin V kit (Cat. No. 88‐8006‐74; Thermo Fisher Scientific). Posttreatment, cells were collected, suspended, and stained with Annexin V‐eFluor 450 and 7‐AAD (15 min, dark, room temperature). Analyze by flow cytometry.

#### 2.2.5 Immunofluorescence

Cells cultured in 24‐well plates were subjected to fixation (4% paraformaldehyde), permeabilization (0.3% Triton X‐100), and blocking (5% BSA). Samples were subsequently incubated with primary antibodies targeting β‐catenin and Wnt5a (1:200; ABclonal) overnight at 4°C, followed by Alexa Fluor 488‐conjugated secondary antibodies (1:500; 1 h) and DAPI nuclear counterstaining. Images were captured via fluorescence microscopy.

#### 2.2.6 ATP Assay

Intracellular ATP levels were measured using an ATP assay kit (S0026; Beyotime Biotechnology, Shanghai, China) according to the manufacturer's instructions. Briefly, after treatment, HK‐2 cells were lysed, and the cell lysates were centrifuged. The supernatant was then collected and mixed with the ATP detection working solution. Luminescence was immediately measured using a SPARK microplate reader (Tecan, Austria). The ATP concentration for each sample was normalized to the total protein concentration as determined by a BCA assay.

#### 2.2.7 Oxygen Consumption Rate (OCR) Assay

OCR was quantified using an Enhanced OCR Fluorometric Assay Kit (Elabscience, Wuhan, China). HK‐2 cells were seeded into black 96‐well microplates at a density of 1 × 10^4^ cells/well. Following the indicated treatments, cultures were incubated with the probe‐containing working solution per the manufacturer's protocol. Kinetic fluorescence was immediately measured at 37°C (excitation: 405 nm, emission: 650 nm) every 2 min for 90 min via a SPARK microplate reader (Tecan, Austria). The OCR (fluorescence units/min) was calculated from the linear slope of the fluorescence‐time curve.

### 2.3 Animal Experiments

KKAy mice are a genetic mutant diabetic mouse model that closely mimics the manifestations of human Type 2 diabetes, making them an ideal animal model for the disease. Eight‐week‐old male KKAy mice (*n* = 12) were used to establish a Type 2 DN model, and age‐matched C57BL/6J male mice (*n* = 6) were used as the normal control group. All the mice were obtained from Beijing Huafukang Biotechnology Co. Ltd. All animal protocols were approved by the Experimental Animal Ethics Committee of the Jilin Academy of Traditional Chinese Medicine (Approval No. JLSZKYDWLL2024‐045) and complied with national care guidelines. Following the acclimatization period, mice in the treatment group were administered Huaier (5 g/kg) once daily via intragastric gavage for 12 weeks. The control and diabetic groups received equal volumes of distilled water. Each group included six mice. Posttreatment, body weights were recorded, and urine was collected for biochemical analysis. Blood samples were obtained under isoflurane anesthesia, subsequently followed by euthanasia via CO_2_ inhalation. Kidneys were then harvested for molecular biology experiments. After the experiment, all animal carcasses were collected for harmless disposal.

#### 2.3.1 Biochemical Analysis of Blood and Urine Samples

Blood samples were centrifuged to separate the serum. Levels of serum creatinine (Scr), urinary albumin, and urinary creatinine were quantified using commercial kits (Nanjing Jiancheng Bioengineering Institute, China) per manufacturer protocols. Subsequently, the urinary albumin‐to‐creatinine ratio (UACR) was derived.

#### 2.3.2 Periodic Acid–Schiff (PAS) Staining

Renal tissues were fixed in 4% paraformaldehyde, dehydrated, embedded in paraffin, and cut into 2‐μm sections. Following deparaffinization and rehydration, sections were stained with Schiff reagent for 15 min and counterstained with hematoxylin. Subsequently, slides were dehydrated, mounted, and imaged via light microscopy.

#### 2.3.3 Transmission Electron Microscopy (TEM)

Kidney samples were fixed in 2.5% glutaraldehyde at 4°C for at least 2–4 h and then postfixed in 1% osmium tetroxide. After dehydration through a graded ethanol series, the tissues were permeabilized with acetone and embedded in epoxy resin. Ultrathin sections (50 nm) were stained with uranyl acetate and lead citrate, and they were imaged via transmission electron microscopy (JEOL, Tokyo, Japan).

#### 2.3.4 TUNEL Assay

Renal apoptosis was assessed using a TUNEL Apoptosis Detection Kit (Alexa Fluor 488; Yeasen, Shanghai, China) per manufacturer protocols. Paraffin sections were deparaffinized, rehydrated, and permeabilized with proteinase K (10 min, 37°C). Samples were incubated with the TUNEL reaction mixture in a dark, humidified chamber (60 min, 37°C). Following DAPI counterstaining and mounting, fluorescent images were captured via fluorescence microscopy.

### 2.4 Network Pharmacology Analysis of Huaier in DN

Active compounds of Huaier were identified by searching the Chemistry Database of the Shanghai Institute of Organic Chemistry, HERB, and BATMAN‐TCM 2.0, supplemented by literature‐derived data. Compounds were screened for oral bioavailability (OB ≥ 20%) and drug‐likeness (DL ≥ 0.1), with putative targets predicted via the TCMSP database. Simultaneously, DN‐related targets were retrieved from GeneCards (relevance score ≥ 10), OMIM, and TTD. To identify hub genes, a protein–protein interaction (PPI) network was constructed via the STRING database (confidence > 0.4), and core targets were determined using the CytoNCA plugin based on topological centrality (degree, closeness, and betweenness). Finally, Gene Ontology (GO) and Kyoto Encyclopedia of Genes and Genomes (KEGG) pathway enrichment analyses were performed using the DAVID platform (*p* < 0.05) to elucidate the biological processes (BP), cellular components (CC), and molecular functions (MF) associated with Huaier's therapeutic effects.

### 2.5 Western Blot Analysis

Total protein was extracted from kidney tissues or HK‐2 cells. Protein concentrations were quantified via a BCA assay (Beyotime, Shanghai, China). Equal amounts of protein (20–40 μg per lane) were separated via SDS–PAGE and transferred onto PVDF membranes. The membranes were blocked and incubated overnight at 4°C with the following primary antibodies: Bcl‐2 (1:1000; Abcam), Bax (1:1000; ABclonal), cleaved‐caspase 3 (1:1000; Cell Signaling), caspase 3 (1:1000; Cell Signaling), p‐GSK3β (1:1000; Cell Signaling), GSK3β (1:1000; Cell Signaling), β‐catenin (1:1000; ABclonal), Wnt5a (1:1000; ABclonal), GAPDH (1:5000; ABclonal), and β‐actin (1:2000; Abcam). Following a series of washes, membranes were incubated with horseradish peroxidase (HRP)‐linked secondary antibodies. Visualization was achieved via chemiluminescence, and relative protein expression was analyzed using ImageJ software.

### 2.6 Statistical Analysis

Data are expressed as mean ± SD from independent triplicates (*n* ≥ 3). Statistical analyses were computed using GraphPad Prism 9.0, employing one‐way ANOVA and Tukey's post hoc correction to assess significance (*p <* 0.05).

## 3 Results

### 3.1 Huaier Alleviates Renal Injury in KKAy Diabetic Mice

To evaluate the renoprotective effects of Huaier, KKAy diabetic mice were treated with Huaier for the indicated duration (Figure 1A). Compared with C57BL/6J mice, diabetic KKAy mice had much greater body weights and blood glucose levels after 20 weeks. Compared with untreated KKAy mice, Huaier‐treated mice presented lower body weights and blood glucose levels (Figure 1B,C). KKAy mice presented a lower kidney‐to‐body weight ratio, and Huaier treatment restored this ratio (Figure 1D). The UACR and Scr levels were high in KKAy mice, indicating kidney damage. Huaier administration significantly reduced both the UACR and the Scr level (Figure 1E,F). Histopathological staining (Figure 1G) revealed that kidneys from control mice had a normal glomerular size and structure, with no mesangial proliferation in the glomeruli or vacuolar degeneration in the tubular epithelial cells. KKAy diabetic mice presented enlarged glomeruli, pronounced mesangial matrix expansion, and marked vacuolar changes in tubular cells. The Huaier‐treated group showed only mild glomerular enlargement, limited mesangial proliferation, and reduced vacuolar degeneration. TEM (Figure 1H) revealed orderly podocyte foot processes and an intact glomerular basement membrane (GBM) in the controls, which contrasted sharply with the extensive foot process fusion and marked GBM thickening in the diabetic group. Huaier treatment partially reversed this glomerular damage, reducing GBM thickening and restoring the architecture of some foot processes. Regarding the renal tubules, control group tubular epithelial cells exhibited normal morphology, intact mitochondrial membrane structure, and orderly mitochondrial cristae. In contrast, diabetic group tubular cells showed marked mitochondrial swelling, with disordered, fragmented, or even disappeared cristae. These pathological alterations were notably ameliorated in the Huaier‐treated group.

**FIGURE 1:**
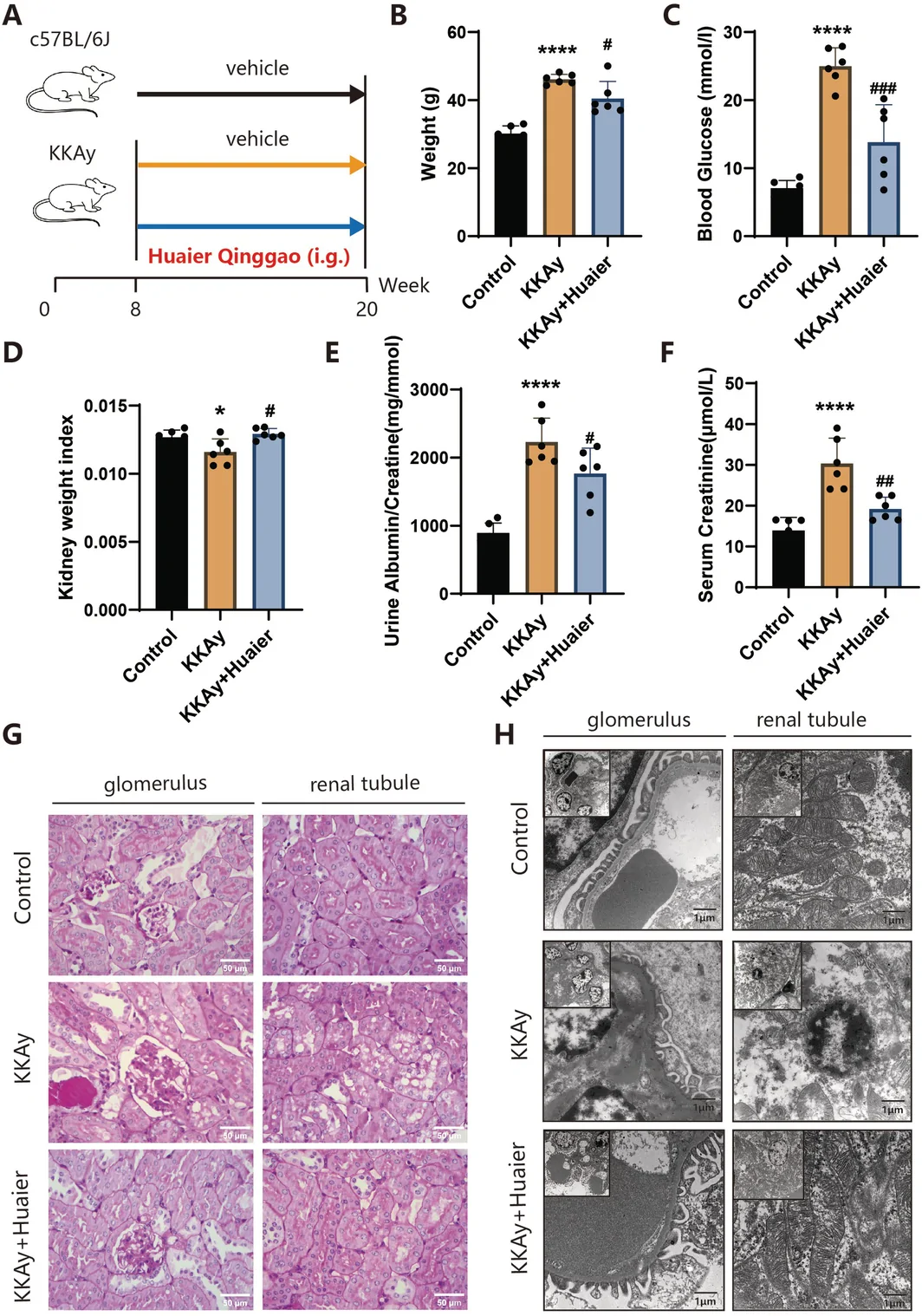
Effects of Huaier on renal function in KKAy mice. (A) Schematic representation of the experimental protocol. (B–D) Assessment of physiological indices: Body weight, blood glucose, and kidney weight index. (E–F) UACR and Scr. (G) Representative PAS‐stained kidney sections; scale bar: 50 μm. (H) Representative TEM images showing ultrastructural changes in mouse kidneys at ×10 000 magnification (scale bar: 2 μm) and ×25 000 magnification (scale bar: 1 μm).

### 3.2 Huaier Prevented RTECs Apoptosis in Kidney Tissue of KKAy Mice

To further investigate the mechanism by which Huaier improves DN, we predicted 851 DN targets using GeneCards (correlation score ≥ 10), OMIM, and TTD databases, and identified 76 Huaier targets from the TCMSP database. We then identified 30 potential targets for Huaier in the treatment of DN (Figure 2A). We performed GO functional enrichment analysis on the 30 targets, and the BP results showed that these targets were primarily concentrated in the apoptosis pathway (Figure 2D). Based on topological parameters, CytoNCA analysis identified 12 core targets, among which CASP3, BCL2, and GSK3B are directly involved in the apoptosis regulation process (Figure 2B,C). TUNEL staining demonstrated significantly reduced apoptotic cell density in Huaier‐treated mice relative to untreated KKAy controls (Figure 2E). Furthermore, Western blot analysis demonstrated that Huaier treatment reversed the pathological decrease in the Bcl‐2/Bax ratio and the elevation of cleaved caspase‐3 observed in KKAy mice (Figure 2F). These results indicate that Huaier alleviates apoptosis in the kidney tissue of KKAy mice.

**FIGURE 2:**
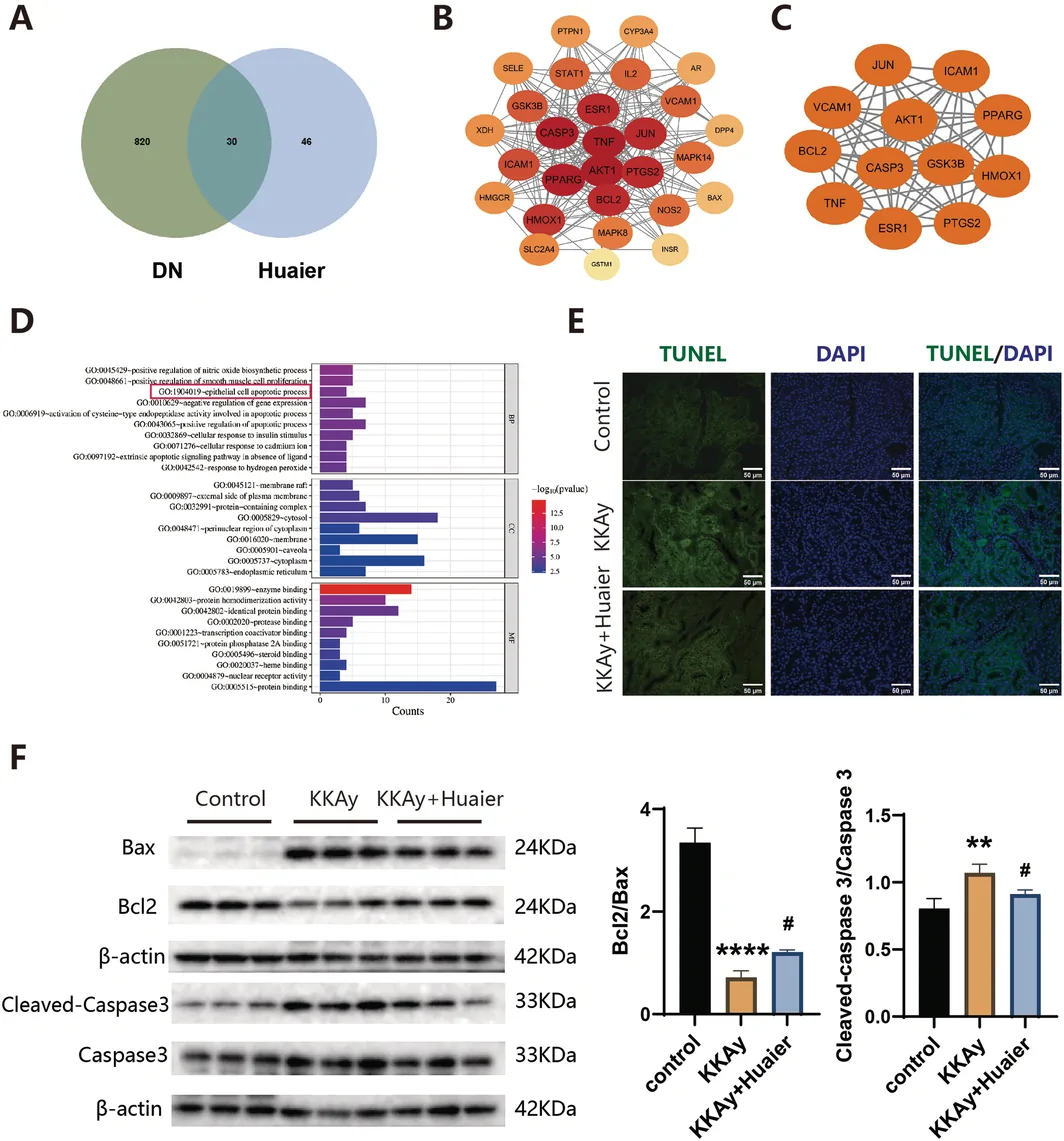
Huaier prevented RTECs apoptosis in KKAy mice. (A) Venn diagram of intersecting targets between Huaier and DN. (B) Protein–protein interaction (PPI) network of candidate targets for Huaier in DN treatment. Node color depth reflects node importance—darker red indicates higher centrality. (C) Top 12 core targets screened by the CytoHubba plugin. (D) Distribution of the top 10 GO terms across BP, CC, and MF categories. (E) Representative images of TUNEL staining showing apoptotic cells in renal tissues. Green fluorescence indicates TUNEL‐positive cells, and blue fluorescence (DAPI) indicates nuclei. Scale bar = 50 μm. (F) Representative Western blots and quantitative analyses of apoptosis‐related proteins, including the Bcl‐2/Bax ratio and cleaved caspase‐3/caspase‐3, in renal tissues from the indicated groups (*n* ≥ 3).

### 3.3 Huaier Protects HK‐2 Cells From High Glucose and Palmitic Acid (HG/PA)‐Induced Cellular and Mitochondrial Injury

To further investigate the protective effects of Huaier at the cellular level, we established a high glucose and palmitate (HG/PA)‐induced injury model in human RTECs (HK‐2). To determine the optimal concentration of Huaier for cell experiments, the effects of various concentrations and durations of exposure to Huaier on HK‐2 cell viability were evaluated via a CCK‐8 assay. Compared with that of the control group, Huaier concentrations ranging from 0.5 to 5 mg/mL had no significant effect on cell viability, whereas cell viability significantly decreased after treatment with Huaier at concentrations greater than 6 mg/mL (*p <* 0.05; Figure 3A). Furthermore, in HG/PA‐treated cells, Huaier at concentrations of 2, 3, and 4 mg/mL partially restored cell viability.

**FIGURE 3:**
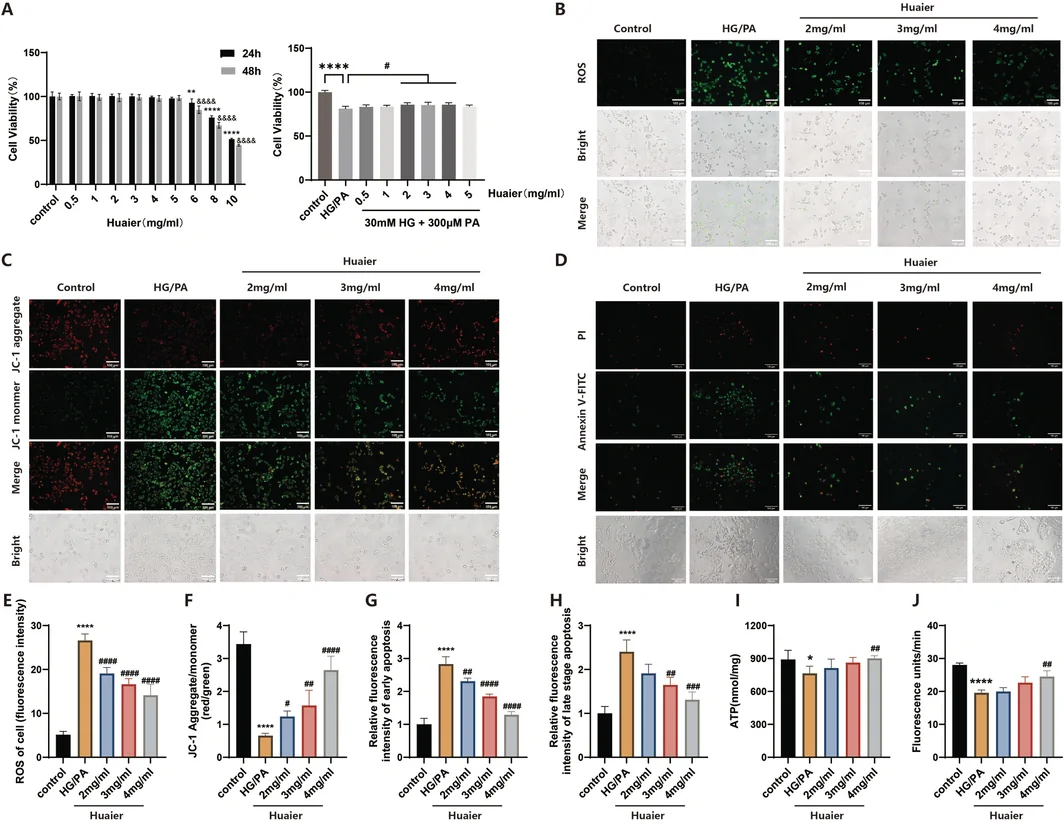
Protective effects of Huaier on HK‐2 cells treated with HG/PA. (A) The viability of HK‐2 cells under different Huaier treatment conditions was assessed by a CCK‐8 assay (*n* = 5). (B) Representative fluorescence microscopy images showing the accumulation of intracellular ROS under various treatment conditions. Scale bar = 100 μm. (C) Representative fluorescence microscopy images of the MMP detected by JC‐1 staining after different treatments. Scale bar = 100 μm. (D) Representative fluorescence microscopy images of Annexin V‐FITC/PI staining. Scale bar = 100 μm. Cell states are defined as: Live (Annexin V^−^/PI^−^); Early Apoptotic (Annexin V^+^/PI^−^); Late Apoptotic/Necrotic (Annexin V^+^/PI^+^); and Dead (PI^+^). (E–H) Quantitative analysis of fluorescence intensity. (I, J) Quantitative analysis of the intracellular ATP concentration and the OCR. The data are presented as the means ± SDs; *n* ≥ 3. **p < 0.01, ***p < 0.001, and ****p < 0.0001 versus the control group; *#p <* 0.05, ##*p <* 0.01, ###*p <* 0.001, and ####*p <* 0.0001 versus the HG/PA group; &&&&*p <* 0.0001 versus the 48 h treatment control group.

To evaluate mitochondrial function, we measured ROS levels, mitochondrial membrane potential (MMP), and intracellular ATP content. As shown in Figure 3B,E, Huaier inhibited ROS accumulation in a dose‐dependent manner in HK‐2 cells. JC‐1 staining, which is used to assess the MMP, revealed a dose‐dependent shift from green to red fluorescence in the Huaier‐treated cells, indicating the mitigation of HG/PA‐induced mitochondrial damage (Figure 3C,F). Furthermore, we assessed key indicators of mitochondrial energy metabolism. We observed that the significant depletion of intracellular ATP (Figure 3I) and the suppression of the cellular OCR (Figure 3J), both of which were induced by HG/PA stimulation, were effectively reversed by treatment with 4 mg/mL Huaier. Finally, Annexin V‐FITC/PI staining revealed that HG/PA markedly induced apoptosis in HK‐2 cells, whereas treatment with Huaier at 2, 3, and 4 mg/mL markedly reduced the number of apoptotic cells, with 4 mg/mL showing the most pronounced effect (Figure 3D,G,H). On the basis of these findings, subsequent experiments were conducted with a Huaier concentration of 4 mg/mL for 24 h.

### 3.4 Huaier Attenuates Apoptosis in HG/PA–Induced HK‐2 Cells

The observed improvements in cell viability and mitochondrial function suggested that Huaier might exert its protective effects by modulating apoptosis‐related pathways. We therefore evaluated the expression of key apoptosis‐related proteins in HG/PA‐treated HK‐2 cells. Compared with control cells, treatment of HK‐2 cells with HG/PA markedly increased cleaved caspase 3 expression but decreased the Bcl‐2/Bax ratio (Figure 4A–D). These alterations were reversed upon treatment with Huaier. To quantitatively confirm the antiapoptotic effect of Huaier, we performed flow cytometry analysis. The apoptotic rate of HK‐2 cells significantly increased following HG/PA stimulation, an effect that was reversed by Huaier treatment (Figure 4E–F).

**FIGURE 4:**
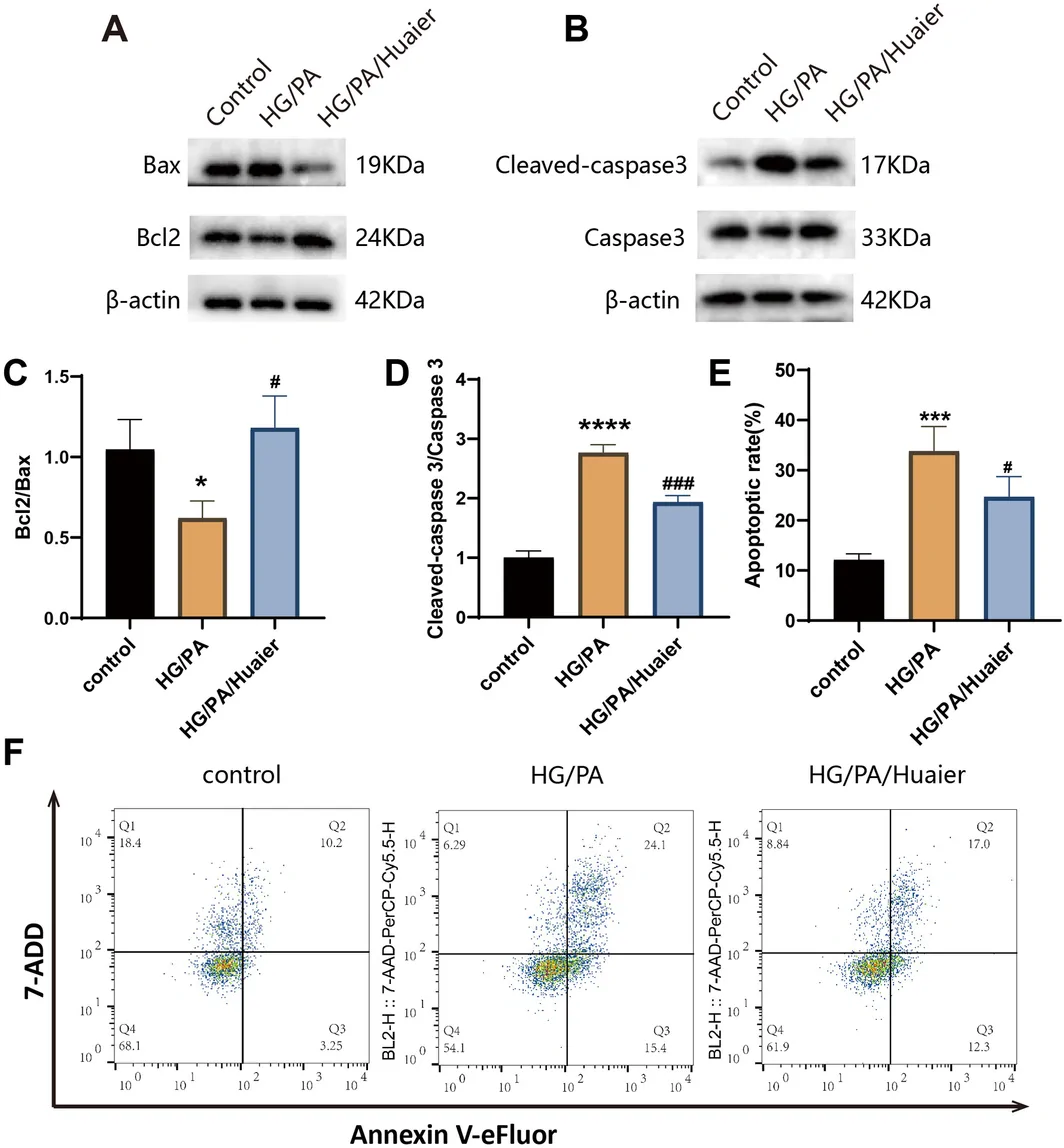
Effects of Huaier on apoptosis‐related protein expression and apoptotic cell proportion in HK‐2 cells exposed to high glucose and palmitate. (A–D) Representative Western blots and quantitative analysis of the protein expression levels of Bcl‐2/Bax, cleaved‐caspase 3/caspase 3 in HK‐2 cells (*n* ≥ 3). (E–F) Flow cytometric assessment of apoptosis in HK‐2 cells via Annexin V/7‐AAD staining.

### 3.5 Huaier Modulates Wnt/β‐Catenin Signaling in HG/PA‐Induced HK‐2 Cells

Given the role of the Wnt/β‐catenin signaling pathway in regulating apoptosis, we next examined whether Huaier exerts its antiapoptotic effects through modulation of this pathway in HG/PA‐treated HK‐2 cells. Western blot analysis demonstrated that HG/PA stimulation aberrantly upregulated β‐catenin and Wnt5a while suppressing p‐GSK3β; notably, Huaier treatment effectively reversed these pathological alterations (Figure 5A–C, *p* < 0.05). Furthermore, immunofluorescence analysis revealed strong expression of β‐catenin in both the nucleus and the cytoplasm of HG/PA‐treated HK‐2 cells. Following Huaier treatment, β‐catenin expression was reduced compared with that in the HG/PA group, and it was predominantly localized in the cytoplasm (Figure 5D–F).

**FIGURE 5:**
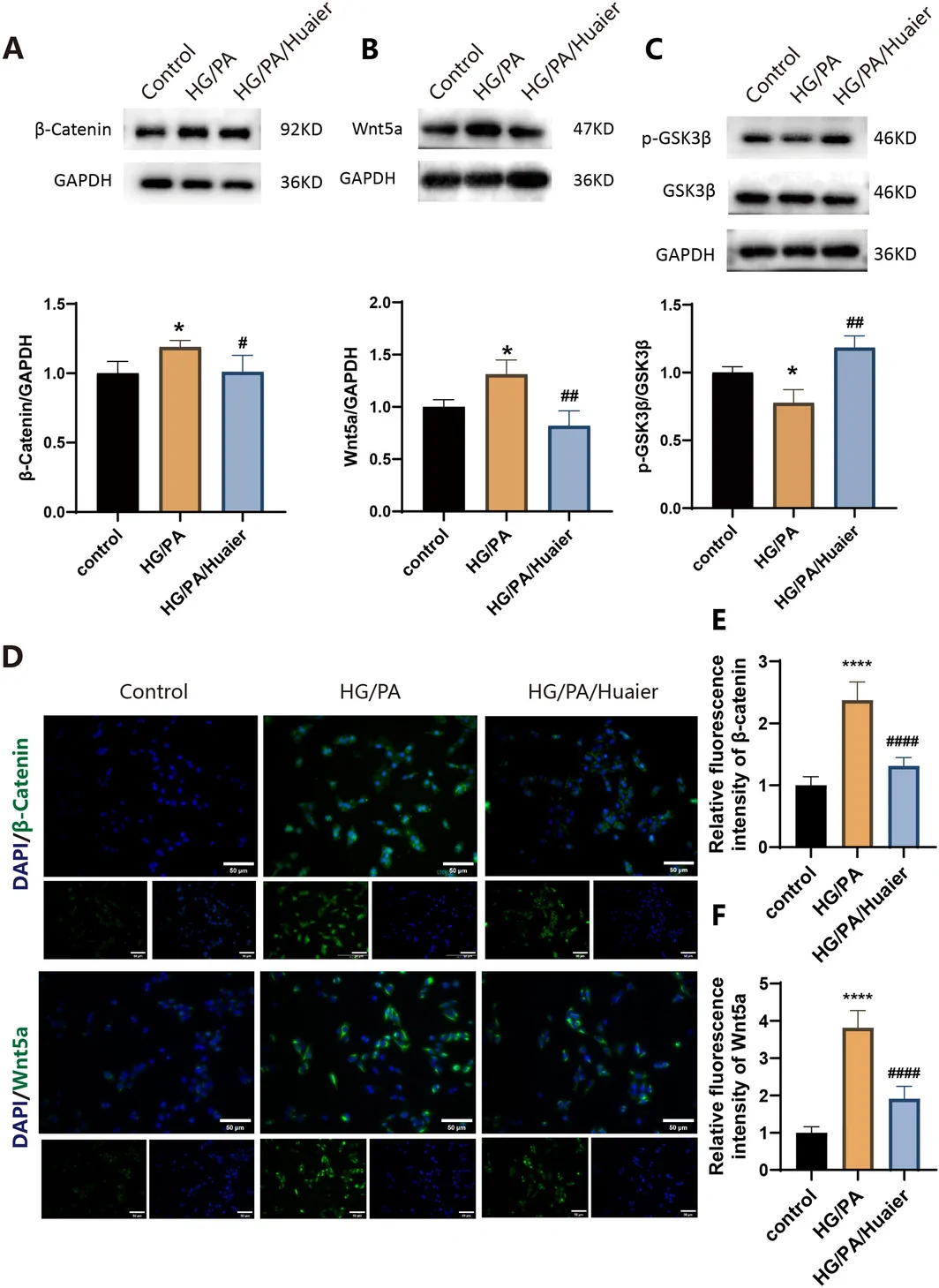
Effects of Huaier on Wnt/β‐catenin–related protein expression and localization in HK‐2 cells under HG/PA conditions. (A–C) Representative Western blots and quantitative analysis of the protein expression levels of p‐GSK‐3β/GSK3β, β‐catenin, and Wnt5a in HK‐2 cells (*n* ≥ 3). (D–F) Immunofluorescence localization and quantification of β‐catenin and Wnt5a (Scale bar: 50 μm; *n* = 6). The data are presented as the means ± SDs. *p < 0.05 and ****p < 0.0001 versus the control group; #*p <* 0.05, ##*p <* 0.01, and ####*p <* 0.0001 versus the HG/PA group.

To confirm the involvement of Wnt/β‐catenin signaling in the protective effects of Huaier, we employed SKL2001, a specific Wnt/β‐catenin agonist, to assess whether activation of this pathway could negate Huaier‐mediated benefits. Compared to the Huaier treatment group, the addition of SKL2001 re‐elevated β‐catenin, Wnt5a, and pro‐apoptotic markers (Bax, cleaved caspase‐3) while suppressing Bcl‐2 (Figure 6A–D).

**FIGURE 6:**
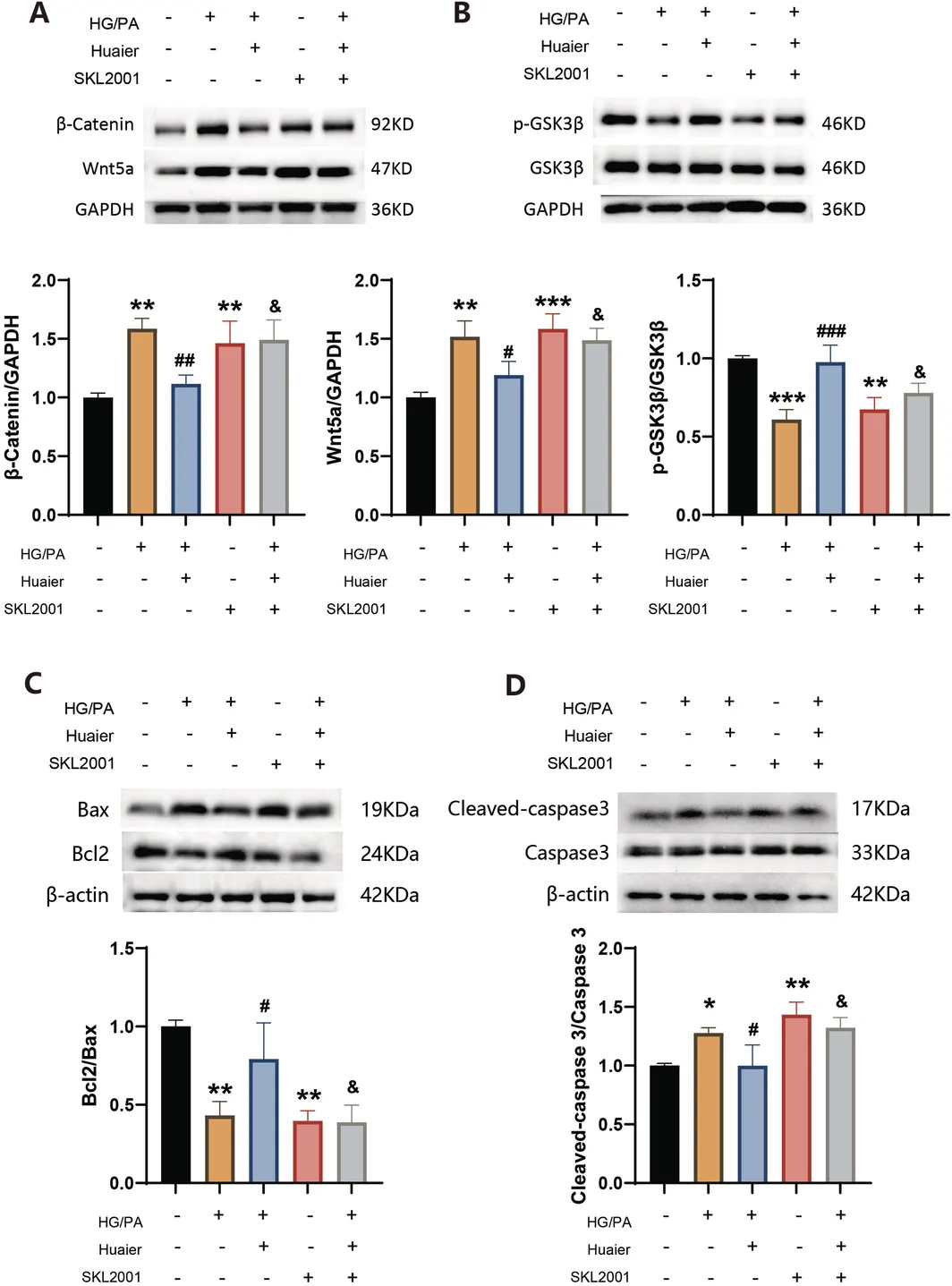
Activation of Wnt/β‐catenin signaling by SKL2001 partially reverses the protective effects of Huaier in HG/PA‐treated HK‐2 cells. (A–D) Impact of Wnt agonist SKL2001 on the expression of Wnt/β‐catenin pathway and apoptotic proteins (*n* ≥ 3). The data are presented as the means ± SDs. *p < 0.05, **p < 0.01, ***p < 0.001 versus the control group; #*p <* 0.05, ##*p <* 0.01, ###*p <* 0.001, and ####*p <* 0.0001 versus the HG/PA group; &*p <* 0.05 and &&*p <* 0.01 versus the HG/PA/Huaier group.

## 4 Discussion

Diabetic nephropathy represents a prevalent microvascular complication of diabetes mellitus that imposes a substantial public health burden, severely compromising both patient quality of life and long‐term survival [18]. Although current therapeutic strategies—such as hypoglycemic and antihypertensive agents—are somewhat effective, their efficacy remains limited and is often accompanied by considerable side effects. Therefore, the development of novel treatment strategies with multitarget regulatory capabilities is urgently needed [19]. Huaier (*Trametes robiniophila* Murr.), a medicinal fungus, contains bioactive components, including polysaccharides, amino acids, and aqueous components. These compounds exhibit diverse biological activities, including anti‐inflammatory and antioxidant effects [12]. Huaier has therapeutic potential in renal diseases, especially in alleviating kidney injury, modulating immune responses, and suppressing inflammation. Previous studies have reported that HGH, a Huaier‐derived compound formulation, significantly ameliorates cisplatin‐induced acute kidney injury by reducing the release and nucleocytoplasmic translocation of high‐mobility group box 1 (HMGB1), thereby inhibiting the TLR4/NF‐κB signaling pathway [20]. Moreover, Huaier suppresses macrophage infiltration in various renal disease models. In an adriamycin‐induced nephropathy model, Huaier alleviates chronic inflammation and podocyte injury by regulating immune cell infiltration and activity [21]. Huaier has also demonstrated therapeutic efficacy in IgA nephropathy and primary nephrotic syndrome, where it alleviates proteinuria and hematuria in patients with mild IgA nephropathy and inhibits mesangial cell proliferation [22]. Moreover, Huaier has shown remarkable nephroprotective effects against chemotherapy‐induced nephrotoxicity. HQH protects renal function without compromising the antitumor efficacy of chemotherapeutic agents, making it a promising adjunct therapy to prevent drug‐induced nephrotoxicity in cancer patients. Although the clinical role of Huaier in the treatment of various cancers and kidney diseases has been established [16, 23, 24], the specific therapeutic role and mechanisms of Huaier in DN remain largely unclear.

Apoptosis is considered one of the pivotal pathological mechanisms in DN, contributing to structural damage to both renal tubules and glomeruli. The accumulation of apoptotic cells not only directly disrupts renal architecture but also promotes excessive extracellular matrix (ECM) deposition, tubular hypertrophy, and mesangial expansion, thereby exacerbating kidney injury [25]. These pathological alterations drive the progression of tubulointerstitial fibrosis and glomerulosclerosis, ultimately leading to a decline in renal function [26]. In the context of DN, hyperglycemia‐induced generation of ROS and reactive nitrogen species further exacerbates renal inflammation, thereby accelerating apoptosis and impairing kidney function [27, 28]. Thus, reducing oxidative stress triggered by hyperglycemia and modulating apoptosis‐related signaling pathways have substantial therapeutic potential in delaying DN progression. Given the susceptibility of tubules to such stress, targeting RTECs' apoptosis has emerged as a highly promising therapeutic strategy. Extensive pharmacological evidence indicates that TCM bioactive components effectively inhibit high glucose‐induced RTECs apoptosis via signaling cascades such as PI3K/Akt and NF‐κB [29, 30]. Notably, compounds like quercetin have been confirmed to suppress apoptosis and restore mitochondrial function specifically in HK‐2 cells. Consistent with these precedents, we selected HK‐2 cells as the in vitro model to elucidate the molecular mechanisms of Huaier.

Under the hyperglycemic conditions characteristic of DN, the expression of apoptosis‐related genes in RTECs is markedly altered. Specifically, the expression of the Bcl‐2 antiapoptotic gene is downregulated, whereas the expression of the Bax and caspase 3 proapoptotic genes is upregulated [31]. With regard to the antitumor mechanisms of Huaier, previous studies have demonstrated that Huaier induces apoptosis in various cancer cell lines, such as A549 and NCI‐H1650, by modulating the Bax/Bcl‐2 ratio [32]. Glycogen synthase kinase‐3β (GSK3β) functions as a pleiotropic serine/threonine kinase critical for orchestrating cell proliferation, metabolic homeostasis, apoptosis, and stress adaptation. GSK3β acts as a negative regulator of the canonical Wnt/β‐catenin signaling pathway, and it inhibits the translocation of β‐catenin into the nucleus and promotes its degradation, thereby suppressing Wnt pathway activation [33]. Aberrant activation of the Wnt/β‐catenin pathway has been implicated in DN pathogenesis, particularly in podocyte injury, apoptosis, and the development of proteinuria [34, 35]. In the present study, HG/PA stimulation activated the Wnt/β‐catenin pathway in HK‐2 cells, as evidenced by increased β‐catenin protein expression and increased nuclear translocation. Collectively, these findings indicate that aberrant Wnt/β‐catenin signaling is a pivotal driver of tubular injury and apoptosis in DN. Moreover, Huaier treatment markedly inhibited both β‐catenin expression and its nuclear translocation. In vivo experiments confirmed that Huaier markedly attenuated the hyperactivation of Wnt/β‐catenin signaling in KKAy mice, thereby alleviating renal tissue injury and reducing the extent of tubular epithelial cell apoptosis.

In current clinical practice, antihyperglycemic agents, ACE inhibitors, and ARBs are widely used to treat DN. However, their therapeutic efficacy remains limited and is often accompanied by adverse effects [19]. In contrast to these monotherapies, Huaier has multitarget and multipathway pharmacological properties, suggesting its potential for combination therapy with existing drugs. By enhancing insulin signaling, suppressing oxidative stress, and modulating immune responses, Huaier may exert synergistic effects that enhance efficacy while reducing toxicity.

### 4.1 Limitations and Future Perspectives

Although the present study provides theoretical and experimental evidence supporting the potential therapeutic efficacy of Huaier in attenuating renal tubular apoptosis in diabetic kidney disease, several limitations warrant acknowledgment. First, while chromatographic fingerprinting and HPLC‐GPC‐ELSD analyses validated the monosaccharide composition and molecular weight distribution profiles of the Huaier aqueous extract, comprehensive qualitative and quantitative profiling of minor active small‐molecule metabolites and glycopeptide micro‐structures remains to be achieved due to the inherent structural complexity of fungal aqueous extracts. Second, standard‐of‐care clinical agents—such as ACE inhibitors, ARBs, or sodium‐glucose cotransporter 2 inhibitors—were not included as positive control groups, thereby limiting a direct comparative assessment of the relative therapeutic potency of Huaier against current first‐line therapies. Third, questions regarding the bioavailability, potential drug–drug interactions, optimal therapeutic dosage, and the use of a limited animal sample size remain unresolved and need further study. Future studies utilizing high‐resolution LC–MS/MS metabolomics and proteomics are necessary to precisely identify specific active constituents, conduct head‐to‐head comparative and combination therapy evaluations with standard clinical agents, and rigorously assess the in vivo pharmacodynamic profile of Huaier across larger animal cohorts.

## 5 Conclusions

The present study demonstrated that Huaier alleviates renal tubular epithelial cell apoptosis and protects against DN, partly through inhibition of the Wnt/β‐catenin pathway. Both in vivo and in vitro findings supported the therapeutic potential of Huaier. These results provide new evidence for the application of Huaier in DN and offer a basis for future translational research.

## Author Contributions

**Yucan Guan:** writing – original draft, investigation. **Ping Nie:** funding acquisition, writing – review and editing. **Ping Luo:** writing – review and editing. **Xiaoying Bai:** formal analysis. **Xianping Wei:** formal analysis. **Tao Ding:** resource.

## Funding

This study was supported by a grant from the Bethune Program of Jilin University (No. 2025B38).

## Ethics Statement

This study was conducted in accordance with the ethical guidelines for animal research and was approved by the Animal Ethics Committee of Jilin Provincial Academy of Traditional Chinese Medicine (Ethics Approval Number: JLSZKYDWLL2024‐045). All experimental procedures were performed in compliance with relevant institutional and national regulations for the care and use of laboratory animals.

## Consent

The authors have nothing to report.

## Conflicts of Interest

The authors declare no conflicts of interest.

## Supporting information


**Supplementary Figure 1.** (A) Molecular weight distribution analysis of Huaier Qinggao (Huaier aqueous extract). (B) Chromatographic fingerprint of Huaier. 1: arabinose, 2: galactose, 3: glucose, 4: xylose, 5: mannose.
**Supplementary Table 1.** Molecular weight distribution parameters and area percentages of Huaier aqueous extract determined by HPLC‐GPC‐ELSD.

## Data Availability

The data that support the findings of this study are available from the corresponding author upon reasonable request.
