# Supplementary material for: Huaier Attenuates Renal Injury in Type 2 Diabetic Nephropathy by Ameliorating Mitochondrial Dysfunction and Inhibiting Tubular Epithelial Cell Apoptosis
Source: J Diabetes. 2026 Oct 29;18(11):e70290. doi: 10.1111/1753-0407.70290 (PMC13719533; doi:10.1111/1753-0407.70290)
Supplement: Supplementary file 1 [file JDB-18-e70290-s001.docx]

**Supplementary Figure 1.** (A) Molecular weight distribution analysis of Huaier Qinggao (Huaier aqueous extract). (B)Chromatographic fingerprint of Huaier. 1: arabinose, 2: galactose, 3: glucose, 4: xylose, 5: mannose.

**Supplementary Table 1.** Molecular weight distribution parameters and area percentages of Huaier aqueous extract determined by HPLC-GPC-ELSD

| Fraction | Mn (Da) | Mw (Da) | MP (Da) | Mz (Da) | Mz+1 (Da) | Polydispersity (Mw/Mn) | Total Area | Area (%) |
| --- | --- | --- | --- | --- | --- | --- | --- | --- |
| 1 | — | — | — | — | — | — | 1,451,251 | 8.16 |
| 2 | — | — | 753,169 | — | — | — | 1,451,251 | 4.66 |
| 3 | 21,914 | 53,692 | 20,938 | 144,625 | 239,933 | 2.45 | 1,451,251 | 55.11 |
| 4 | — | — | 3,300 | — | — | — | 1,451,251 | 2.22 |
| 5 | — | — | 768 | — | — | — | 1,451,251 | 2.83 |
| 6 | — | — | 373 | — | — | — | 1,451,251 | 11.52 |
| 7 | — | — | 230 | — | — | — | 1,451,251 | 13.63 |
| 8 | — | — | — | — | — | — | 1,451,251 | 1.23 |
| 9 | — | — | — | — | — | — | 1,451,251 | 0.65 |
